# Dry Etching of Copper Phthalocyanine Thin Films: Effects on Morphology and Surface Stoichiometry

**DOI:** 10.3390/molecules170910119

**Published:** 2012-08-24

**Authors:** Jaron G. Van Dijken, Michael J. Brett

**Affiliations:** 1 Department of Electrical and Computer Engineering, University of Alberta, Edmonton, AB T6G 2V4, Canada; 2 NRC-National Institute for Nanotechnology, Edmonton, AB T6G 2M9, Canada

**Keywords:** phthalocyanine dyes, vapor phase processes, dry etching, nanopillar formation

## Abstract

We investigate the evolution of copper phthalocyanine thin films as they are etched with argon plasma. Significant morphological changes occur as a result of the ion bombardment; a planar surface quickly becomes an array of nanopillars which are less than 20 nm in diameter. The changes in morphology are independent of plasma power, which controls the etch rate only. Analysis by X-ray photoelectron spectroscopy shows that surface concentrations of copper and oxygen increase with etch time, while carbon and nitrogen are depleted. Despite these changes in surface stoichiometry, we observe no effect on the work function. The absorbance and X-ray diffraction spectra show no changes other than the peaks diminishing with etch time. These findings have important implications for organic photovoltaic devices which seek nanopillar thin films of metal phthalocyanine materials as an optimal structure.

## 1. Introduction

Ion sputtering is often employed by surface analysis techniques, such as auger electron spectroscopy (AES), X-ray photoelectron spectroscopy (XPS), and sary ion mass spectrometry (SIMS), to generate depth profiles of multilayer films. The limits imposed on depth profiling by the effects of ion sputtering on surface topography have been studied thoroughly for many metal and semiconductor thin films [1,2,3,4,5,6,7,8,9,10,11]. Ripples and cones are among the most common surface features produced by ion bombardment. The severity of surface topography changes during ion sputtering depends on the characteristics of the sample and the ion beam, and can often be reduced by means of sample rotation [5,12,13]. Accurate depth profiling of polymer and organic layers is considerably more challenging than inorganic materials, but has been improved as of late [14,15,16,17,18,19,20]. Few studies have been done on plasma etched metal phthalocyanine (MPc) films, and the effects on morphology are not well known.

This paper investigates the evolution of copper phthalocyanine (CuPc) and zinc phthalocyanine (ZnPc) thin films as they are etched with argon and oxygen plasmas. Significant morphological changes occur as a result of the ion bombardment: A planar surface becomes an array of nanopillars with sub-20 nm diameters. This result is intriguing from an organic photovoltaic perspective, where nanopillar film morphologies are desired whose pillar diameters closely match the short exciton diffusion lengths of around 15 nm seen in organic materials [21]. Many methods have been used to attempt this nanopillar morphology, such as solvent annealing [22], solvent recrystallization [23], organic vapor phase deposition [24], glancing angle deposition [25,26,27,28,29], substrate seeding [30,31], and heat treatments [32,33]. However, nanopillar films remain much less than 100 nm in thickness in most cases, which leaves absorption insufficient for achieving optimal device efficiencies. Therefore, alternative methods are desired for fabricating nanopillar organic films. Plasma etching presents an entirely new approach to structuring organic thin films at the nanoscale. In previous studies, ion bombardment has been found to induce surface texturing on various inorganic materials, which is often explained by the preferential erosion of grain boundaries [2,34,35]. The results presented in this paper exhibit similar behavior, enabling a new approach to nanostructuring MPc thin films.

## 2. Results and Discussion

### 2.1. Columnar MPc Film Morphologies via Plasma Etching

**Figure 1:**
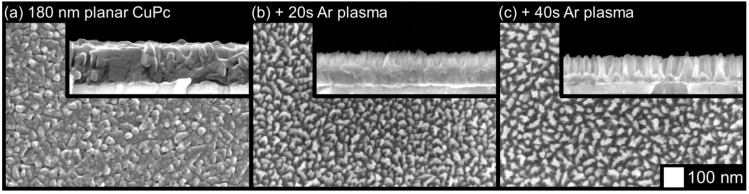
Effects of argon plasma etching on a planar CuPc thin film.

The first nanopillar films were fabricated by etching a set of 180 nm thick planar CuPc films with argon plasma. Films were etched with an RF power of 220 W for 20 and 40 ss, resulting in the columnar morphologies seen in Figure 1. With column diameters of around 20 nm or less, these morphologies are smaller than the columns found in the best small molecule films produced to date [33], and are extremely well suited to the exciton diffusion lengths of MPc materials. In addition, the etched columns in Figure 1c are wide at the base and taper off at the top, which may improve their survivability during the solution-based filling processes commonly used in OPV device fabrication.

**Figure 2:**
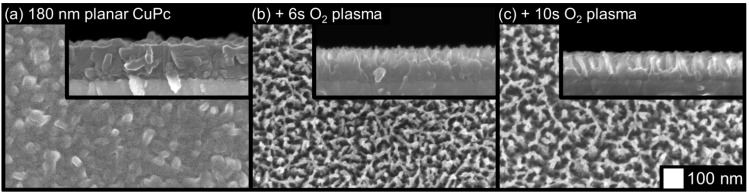
Effects of oxygen plasma etching on a planar CuPc thin film.

Etching with oxygen gas instead of argon was also performed, with a chamber pressure of 150 mTorr, RF power of 220 W, and gas flow rate of 80 sccm. The results are shown in Figure 2. Here, etching proceeded much faster due to the reactivity of ionized oxygen, which enhances removal of organic material in conjunction with physical sputtering. A somewhat similar final morphology can be achieved with oxygen plasma compared to argon plasma, albeit with a more disordered and fragile network of remaining features. The likelihood of chemical changes to the film is higher with oxygen, making oxygen plasma etched films less interesting than argon plasma etched films. As such, argon was determined to be the etching gas of choice, and no additional oxygen-etched films were investigated.

**Figure 3:**
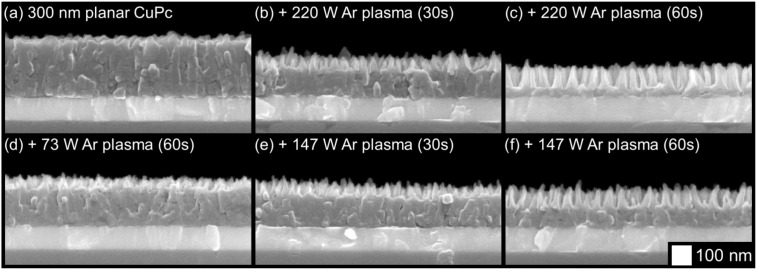
Effects of different etch conditions and etch times on the morphology of CuPc thin films etched with argon plasma.

The RF power was varied to investigate the sensitivity of morphology to the etch conditions. A 300 nm planar film was exposed to argon plasmas for various periods of time with three levels of RF power: 73 W, 147 W, and 220 W. The images shown in Figure 3 reveal similar etching behavior in all cases, only at different rates. Etched films resembled others that were etched to similar depths under different conditions. Thus, the etching mechanism remains the same, and changes in RF power affect the etch rate only.

**Figure 4:**
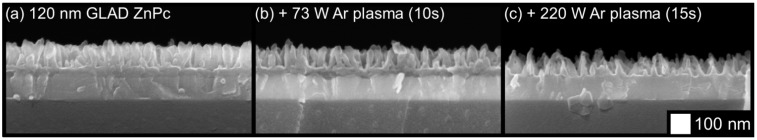
Effects of different argon plasma etching conditions on a thin GLAD ZnPc film.

It may be possible to further improve these etched film morphologies by starting with an already textured MPc film. For example, glancing angle deposition (GLAD) has been used previously to grow nanopillar MPc films suitable for OPV devices [25,26,27,28,29]. GLAD is a physical vapor deposition technique that produces nanopillar film morphologies by placing the substrate at an oblique angle relative to incoming vapor flux [36]. Combining GLAD with plasma etching could introduce additional control over columnar dimensions and spacing, while requiring less etching and disturbance of the original film. Alternatively, this approach can be thought of as a way to refine the structure of a GLAD film via ion bombardment, which is not an entirely new concept. In fact, ion milling and reactive ion etching have been shown previously to enhance the morphology and properties of GLAD films for various inorganic materials in other applications [37,38,39,40,41]. Figure 4 shows the effects of etching a 120 nm GLAD ZnPc film. The original film has a slanted post morphology (pointing into the page), which appears largely untouched by a low power etch but deteriorates significantly under a more aggressive etch (Figure 4b *vs*. 4c), with an overall thinning of the areal density of posts observed. In these cases, any structural enhancements due to etching are not obvious. It is possible that a vertical post morphology may be more receptive to plasma etch refinements, and that thicker films with broader columns would benefit more, but this will require further study.

### 2.2. Surface Analysis and Electronic Properties

The creation of columnar film morphologies out of planar ones with ion sputtering potentially affects the surface composition. The harmful effects of UV radiation from the plasma alone may impact the survivability of the sample [42,43]. In addition, when considering that the ion energies from the plasma are at least comparable to or greater than the intramolecular bond energies in metal phthalocyanines, changes in surface composition may be expected [44,45]. Unsurprisingly, ion beams with MeV ion energies have been shown to be very destructive to MPc molecules [46]. However, the strong π network of MPc molecules may help them avoid fragmentation from impacts of only tens of eV, due to protection by delocalized electrons [47].

**Figure 5:**
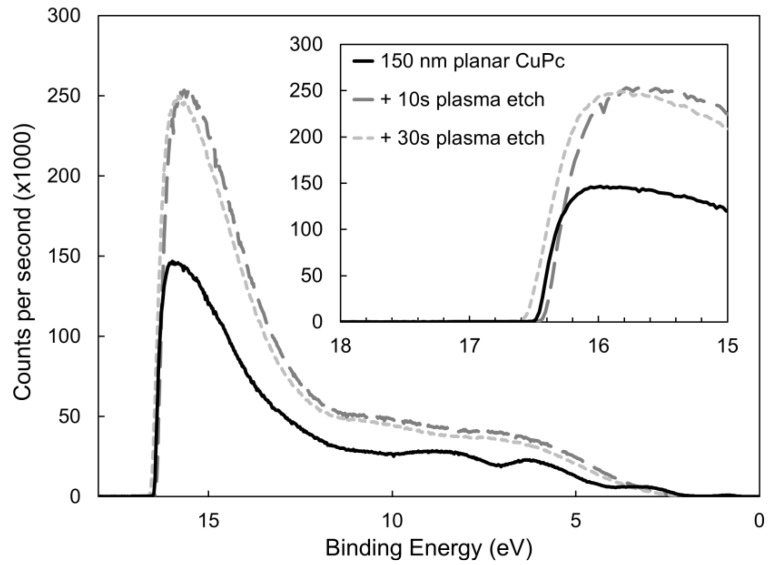
Changes to the UPS spectra of a planar CuPc film due to argon plasma etching.

The surfaces of the etched CuPc films in this investigation were analyzed with ultraviolet photoelectron spectroscopy (UPS) and X-ray photoelectron spectroscopy (XPS). The UPS curves, shown in Figure 5, show virtually no change in the onset energy for electron ejection, at around 16.5 eV. Consequently, the work function is consistent across all samples, at 4.7 ± 0.1 eV, which is slightly higher than other published values [48,49], but may be simply due to more air exposure [50]. These results provide little insight into the effects of etching, and direct contact resistance measurements could not be performed due to the mechanical sensitivity of the nanopillar films. However, analysis by XPS reveals more significant information.

**Figure 6:**
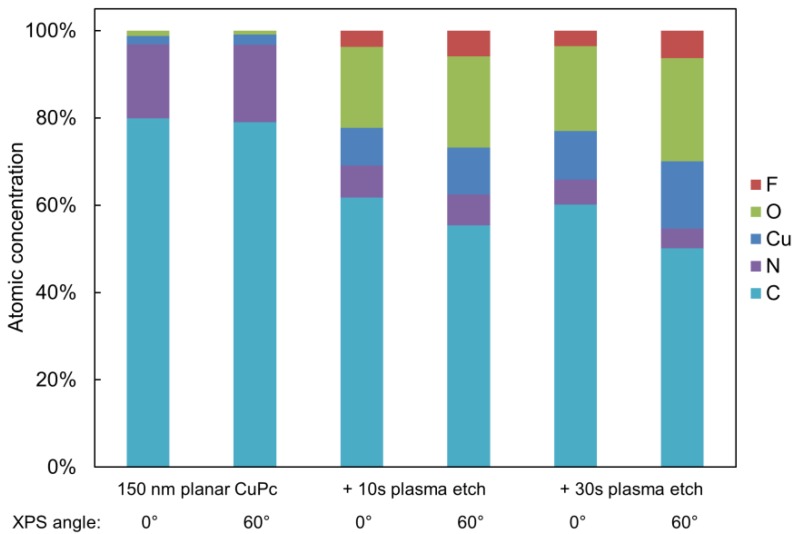
Changes to the surface composition of a planar CuPc film due to argon plasma etching.

The atomic concentrations of various elements were calculated from the relative XPS peak heights, and are presented in Figure 6. Significant and immediate removal of C and N is evident in the sample etched for 10 s, which does not change much further in the 30 s etch. Meanwhile, higher concentrations of Cu are revealed by the etching, which is consistent with results from other etch chemistries [45]. Evidently, the CuPc molecules are not surviving the impacts of the Ar ions, and lighter elements are being preferentially removed over heavier ones. Interestingly, a significant amount of O and F are introduced to the surface, which are likely the result of residual gases in the etch chamber binding to the fragmented molecules as a means of stabilization. For device quality CuPc it may be necessary to perform the etching in a UHV environment. The composition of the original planar CuPc film is independent of the incident beam angle, which suggests that the surface and subsurface are very similar. For the etched films, however, the surface is slightly richer in Cu while being depleted of C and N, which confirms the preferential removal of C and N over Cu mentioned previously. These changes to surface composition may impact potential functionality of the CuPc nanopillars at the active layer interface of an OPV device.

### 2.3. Bulk Film Analysis

**Figure 7:**
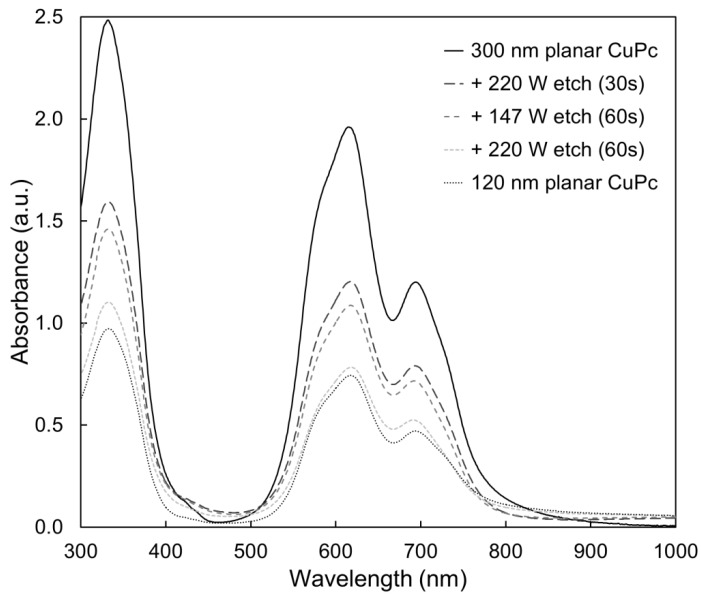
Changes to thin film absorbance as a result of argon plasma etching.

**Figure 8:**
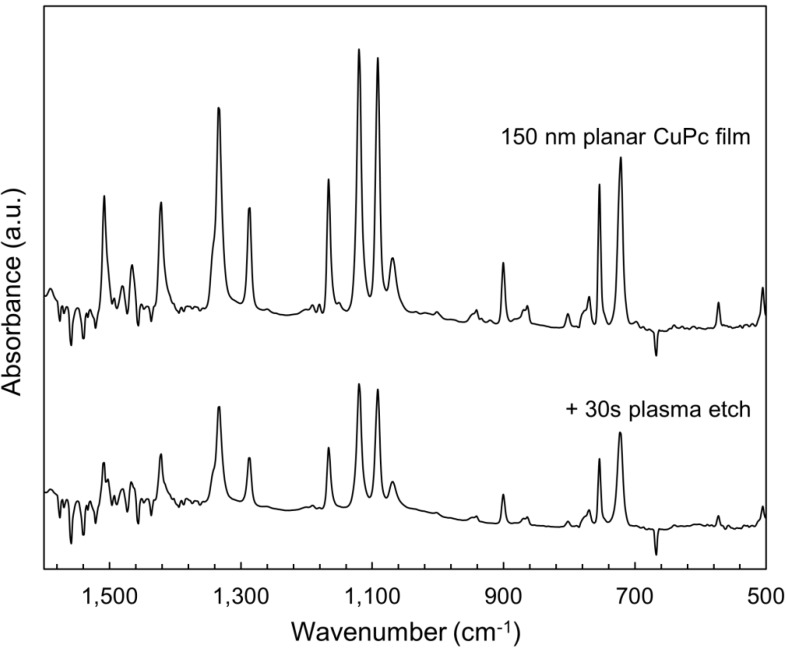
Changes to FTIR absorbance spectra of a CuPc thin film due to 220 W argon plasma etching.

**Figure 9:**
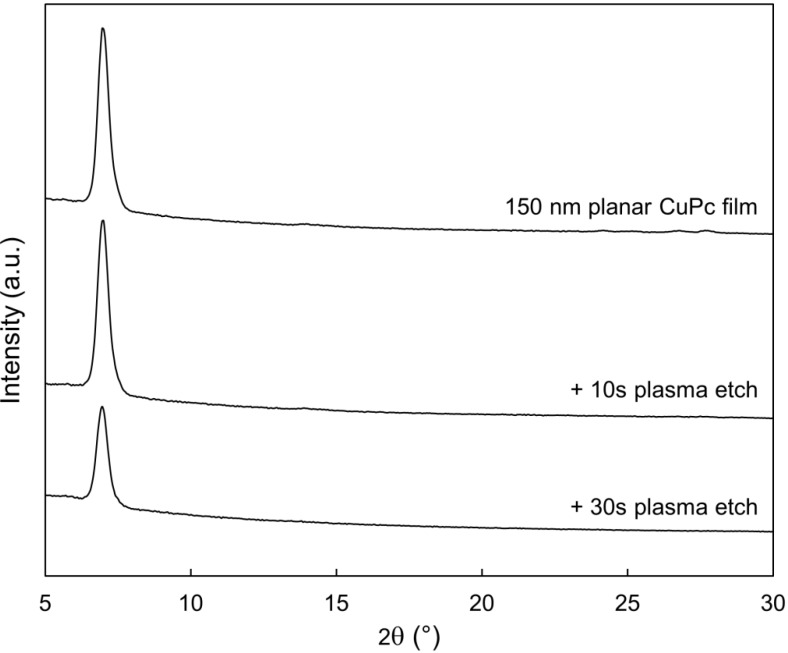
Changes to the XRD spectrum of a planar CuPc thin film as a result of 220 W argon plasma etching.

The surface composition modifications shown in the previous section warrant a study of the bulk properties of the remaining film. Absorbance profiles were gathered in both the visible and infrared spectral regions, which are shown in Figure 7 and Figure 8, respectively. In both cases, no changes to the shape of the absorbance spectra are seen as a result of the etching, but only diminished peak heights. This is consistent with the removal of material seen by SEM, and confirms that much of the remaining film avoids being damaged. Likewise, the X-ray diffraction (XRD) profiles shown in Figure 9 show no changes other than peaks diminishing with etch time. Thus, the material that does remain in the film appears to maintain both its molecular and crystalline structure. It is significant to note that the most etched CuPc film in Figure 7 maintains comparable absorbance to a 120 nm planar un-etched film. Therefore, these films possess nearly the same amount of CuPc material, equivalent to six times the amount used in a typical bilayer OPV device [21]. This confirms that the film morphology seen in Figure 3c is suitable as an ideal OPV active layer architecture. Every point of light absorption in this nanopillar film would be within a single exciton diffusion length of the donor/acceptor interface, and the film would capture significantly more incident light than a commonly used 20 nm planar layer. If the interface were functional, the architecture fabricated by plasma etching would be very useful.

Alternative sputtering techniques may enable structuring of MPc films while minimizing surface damage. Recently, C_60_ ion beams have been used to minimize sputtering damage to organic films during analysis [16,17,18,19,20]. Increasing the incident angle of the ion beam has been found to further minimize sputtering damage [20].

Coincidentally, C_60_ is the acceptor molecule commonly used with MPc materials in the active layer of small molecule OPV devices. This may be very suitable if ion energies are used that are conducive to maintaining molecular structure, particularly since any residue in the film from the ion beam may be functional in the active layer. Therefore, attempting damage-free, ion-induced structuring of MPc films with C_60_ is worthy of further investigation.

## 3. Experimental

CuPc and ZnPc were purchased from Sigma-Aldrich (Oakville, ON, Canada) and purified via thermal gradient sublimation prior to deposition. Both planar and GLAD thin films were deposited in a standard GLAD apparatus [36] via thermal evaporation, with a throw distance of 34 cm, a source temperature of about 400 °C, a base pressure of 2 × 10^−7^ Torr, and a deposition rate of 0.7 Ǻ/s normal to the surface. A variety of substrates were used, to enable various characterization techniques. ITO-coated glass (Delta Technologies, 8–12 Ω/®) was used for imaging via SEM; Si wafers (Evergreen Semiconductor Materials) were used for analysis by XRD, XPS, and UPS; KBr discs (Fisher Scientific) were used to gather FTIR profiles; and glass microscope slides (Fisher Scientific) were used for UV/Vis absorbance profiles. Argon plasma etching was performed in an Oxford Plasmalab μEtch RIE chamber, with 110 sccm Ar gas flow and a chamber pressure of 190 mTorr. The standard RF power used was 220 W, but was changed on occasion, as explained in the text.

Electron microscope images were acquired using a Hitachi field emission S-4800 SEM. XPS was performed at high vacuum (<5 × 10^−10^ Torr) with a Kratos Ultra spectrometer, using a Al Kα radiation source (hν = 1,486.71 eV). Measurements were done using incident beam angles of 0° and 60° to probe different surface depths. Atomic concentrations were calculated using the relative peak intensities while accounting for the atomic sensitivity factors. UPS was performed in the same environment as XPS, using a He I (hν = 21.21 eV) radiation source. Absorbance over visible regions was acquired as the negative logarithm of transmittance, using a Perkin Elmer Lambda 900 UV/VIS/NIR spectrophotometer. Infrared absorbance was captured with a Nicolet Nexus 760 FTIR spectrometer with a DTGS detector and a sample chamber purged with N_2_. XRD data was collected with a Bruker D8 diffractometer, equipped with a Cu source and area detector, and calibrated against a silver behenate reference standard.

## 4. Conclusions

This paper investigated the effects of argon plasma etching on metal phthalocyanine thin films. Planar films of these materials are readily transformed into nanopillar films with dimensions extremely well suited to OPV devices. The films maintain much of their original molecular characteristics, such as absorbance profiles and crystalline order. However, changes to the surface composition suggest that the ion bombardment causes fragmentation of the molecules at the film surface. Avoiding or repairing this surface damage is necessary to enable functionality in OPV devices, which could provide a new approach to building idealized organic photovoltaic architectures.
